# Serine Proteinases in *Leishmania (Viannia) braziliensis* Promastigotes Have Distinct Subcellular Distributions and Expression

**DOI:** 10.3390/ijms20061315

**Published:** 2019-03-15

**Authors:** Raquel Santos-de-Souza, Luzia Monteiro de Castro Côrtes, Karen dos Santos Charret, Léa Cysne-Finkelstein, Carlos Roberto Alves, Franklin Souza-Silva

**Affiliations:** 1 Fundação Oswaldo Cruz, Instituto Oswaldo Cruz, Laboratório de Biologia Molecular e Doenças Endêmicas, Avenida Brasil, 4365, Manguinhos, Rio de Janeiro 21040-900, Brazil; ra.souza04@gmail.com (R.S.-d.-S.); luzia@ioc.fiocruz.br (L.M.d.C.C.); karenbiologa@gmail.com (K.d.S.C.); franklin.frankss@gmail.com (F.S.-S.); 2 Fundação Oswaldo Cruz, Instituto Oswaldo Cruz, Laboratório de Imunoparasitologia, Avenida Brasil, 4365, Manguinhos, Rio de Janeiro 21040-900, Brazil; lcysne@ioc.fiocruz.br; 3 Fundação Oswaldo Cruz, Centro de Desenvolvimento Tecnológico em Saúde, Avenida Brasil, 4365, Manguinhos, Rio de Janeiro 21040-900, Brazil

**Keywords:** *Leishmania (Viannia) braziliensis*, protease, serine proteinases, subtilisin-like, HiTrap benzamidine column, Z-FR-AMC, Suc-AFK-AMC

## Abstract

Serine proteinases in *Leishmania (Viannia) braziliensis* promastigotes were assessed in this work. This study included the investigation of the enzymatic activity of subcellular fractions obtained from benzamidine affinity chromatography, reverse transcription polymerase chain reactions, and in silico assays of subcellular localization of subtilisin. Promastigote serine proteinases showed gelatinolytic activity with molecular masses of 43 kDa to 170 kDa in the cytosolic fraction and 67 kDa to 170 kDa in the membranous fraction. Serine proteinase activities were detected using *N*-benzyloxycarbonyl-l-phenylalanyl-l-arginine 7-amino-4-methylcoumarin (Z-FR-AMC) and *N*-succinyl-l-alanine-l-phenylalanine-l-lysine 7-amino-4-methylcoumarin (Suc-AFK-AMC) as substrates in the cytosolic fraction (Z-FR-AMC = 392 ± 30 µmol.min^−1^ mg of protein^−1^ and Suc-AFK-AMC = 252 ± 20 µmol.min^−1^ mg of protein^−1^) and in the membranous fraction (Z-FR-AMC = 53 ± 5 µmol.min^−1^ mg of protein^−1^ and Suc-AFK-AMC = 63.6 ± 6.5 µmol.min^−1^ mg of protein^−1^). Enzyme specificity was shown by inhibition with aprotinin (19% to 80% inhibition) and phenylmethanesulfonyl fluoride (3% to 69%), depending on the subcellular fraction and substrate. The expression of subtilisin (LbrM.13.0860 and LbrM.28.2570) and tryparedoxin peroxidase (LbrM.15.1080) genes was observed by the detection of RNA transcripts 200 bp, 162 bp, and 166 bp long, respectively. Subsequent in silico assays showed LbrM.13.0860 can be located in the cytosol and LbrM.28.2570 in the membrane of the parasite. Data obtained here show the subcellular distribution and expression of serine proteinases, including the subtilisin-like serine proteinases in *L. (V.) braziliensis* promastigotes.

## 1. Introduction

Leishmaniases are endemic diseases that are widespread in regions such as Asia, Europe, Africa, and the Americas. Due to the fact that they are difficult to control, their capacity to cause epidemics, and their ability to cause fatalities and deformities, these diseases are important public health problems [1]. Among the 53 *Leishmania* species reported, only 20 can cause human infection [2]. Parasite transmission occurs in a vector-host cycle through blood repast of female phlebotomines in the *Lutzomyia* or *Phlebotomus* genus in a vertebrate host [3]. In a vertebrate host, the parasites are phagocytosed by macrophages and differentiate into amastigotes that, after intense multiplication by binary fission, rupture the cells and immediately infect new cells [4]. The appearance of ulcers may occur after the second week of infection [5,6], and after this initial lesion, the progression of the disease depends on several factors, including the *Leishmania* species and the patient’s immune system status. There are traditionally four clinical presentations of the disease: cutaneous leishmaniasis, cutaneous-diffuse leishmaniasis, cutaneous-mucosal leishmaniasis, and visceral leishmaniasis [1].

The biological cycles of different *Leishmania* spp. are strongly driven by nuances in their metabolic profiles that are related to the composition of their specific degradomes, including the protease and its respective substrate repertoire [7]. Their enzyme class (EC) is hydrolases (3) subclassified as peptidases (3.4) according to their physicochemical, biochemical, and structural features. Based on the method of peptide bond cleavage, there are two groups of serine proteinases: exopeptidases (EC 3.4.11-19) that cleave peptide bonds at the ends of a polypeptide, and endopeptidases or proteinases (EC 3.4.21-99), which cleave internal peptide bonds within the polypeptide chain and are named based on the amino acids that form the catalytic site, e.g., aspartic proteinases, cysteine proteinases, metalloproteinases, serine proteinases, threonine proteinases, and glutamic proteinases [8].

Aspartic proteinases, cysteine proteinases, metalloproteinases, and serine proteinases whose actions ensure the survival, proliferation, and maintenance of the *Leishmania* spp. life cycle in the host have been described. These enzymes act as virulence factors implicated in tissue invasion, survival of *Leishmania* sp. in macrophages, and modulation of immune response, driving specific clinical manifestations in the mammalian host [9,10]. Specifically, *Leishmania (Viannia) braziliensis* proteinase genes represent 2.18% of the parasite’s genome. In this parasite, metalloproteinase genes predominate the parasitic protease genes (14 families distributed in 7 clans), followed by cysteine proteinases (11 families distributed in 3 clans), and serine proteinases (10 families distributed in 8 clans). Aspartic proteinases are present in lower abundance in this parasitic genome (2 families distributed in 2 clans) [11].

Of the 17 predicted biological functions related to *Leishmania* sp. serine proteinase genes, only 18% were related to parasite physiology, including their activity as signal peptidases for removing the signal peptide from secretory preproteins, as maturases of other proteins, and as metacaspases [7]. The 26 to 28 serine proteinase genes from *Leishmania* sp. are classified in 10 families (S8, S9, S10, S12, S15, S16, S26, S51, S54 and S59) and grouped into 8 clans (SB, SC, SE, SF, SJ. SP, ST and PC) [7,12]. Unlike metalloproteinases and cysteine proteinases, whose biologic activities have been proven in the life cycle of *L. (V.) braziliensis*, serine proteinases have not been properly studied in this parasite [13].

Serine proteinases interact in a network of other proteases, proteins, and/or enzymes in the parasite [7]. As an example, subtilisin is a maturase of tryparedoxin peroxidase (TXNPx), an enzyme involved in the survival of the parasite inside macrophages, which plays a role in the detoxification system, namely trypanothione reductase system [14]. There are subtilisin genes in chromosomes 13 and 28 in *Leishmania* spp. However, only the genes LinJ13_V3.0940 and LmjF13.1040 have been related with this function [14]. Since these genes are orthologous to serine proteinases in *L.(V.) braziliensis*, it is possible that the Lbrm.13.0860 gene has the same function, but this has not yet been confirmed for the gene in chromosome 28.

This work aimed to study serine proteinases of *L. (V.) braziliensis*, including subtilisin-like proteinases (Family S8, Clan SB). The genome of this parasite contains two subtilisin genes (LbrM.13.0860 and LbrM.28.2570) and one TXNPx gene (LbrM.15.1080). To date, the relationship between these genes and their respective gene products in *L. (V.) braziliensis* has not been adequately described. The present study contributes to knowledge on the subcellular distribution of serine proteinases and the expression of two subtilisins in this parasite.

## 2. Results

### 2.1. Detection of Serine Proteinases in Subcellular Fractions of Promastigotes

In the first step of this study, the subcellular locations of serine proteinase of *L. (V.) braziliensis* were assessed. These assays were performed with serine proteinase-enriched fractions (membrane fraction and cytosolic fraction), obtained by affinity chromatography that were analyzed by using gelatin-SDS-PAGE, fluorogenic peptide substrates, and specific inhibitors.

**Figure 1:**
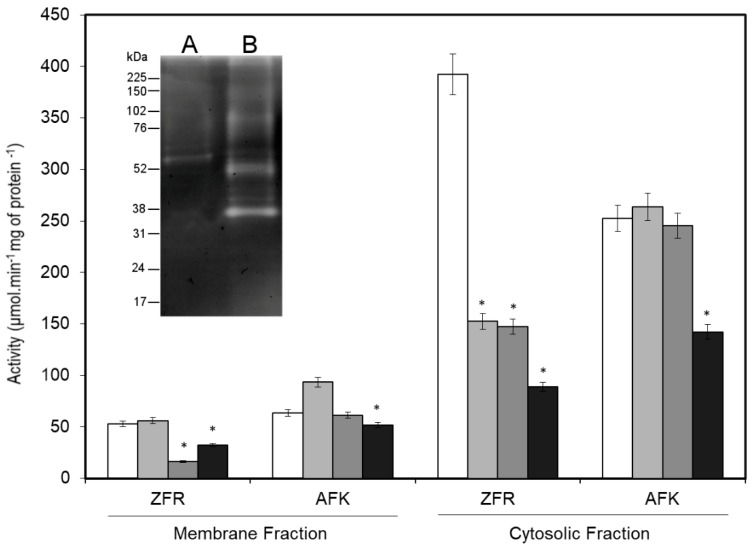
Serine proteinase activity in *Leishmania (V.) braziliensis* promastigote fractions. The enzymatic activity assays in solution were carried out with parasitic proteins (5 μg) from enriched subcellular fractions (membrane and cytosolic) by benzamidine affinity chromatography with Z-FR-AMC or Suc-AFK-AMC (0.1 mM) as the substrate in activation buffer. The activities were assessed without inhibition (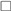) or in presence of 10 μM E-64 (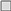), 1 mM PMSF (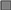) and 5 μg aprotinin (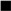). The activities (µmol·min^−1^·mg of protein^−1^) are represented as the average and standard deviation (±) of three independent experiments. Inset, zymographic profile of membrane (**A**), and cytosolic (**B**) fractions enriched with serine proteinase (15 μg). The molecular mass markers are indicated on the left (kDa). These results are representative of three independent experiments. * *p* < 0.05.

Both the membrane and cytosolic fractions were obtained from 10^8^ promastigotes/mL yielding approximately 0.6 ± 0.02 mg/mL protein, which showed a gelatin-SDS-PAGE profile with major proteinase bands with estimated molecular masses of 43 kDa, 48 kDa, 63 kDa, 99 kDa, and 170 kDa in the cytosolic fraction and 67 kDa, 75 kDa, and 170 kDa in the membrane fraction (Figure 1 inset).

The specific substrates Z-FR-AMC and Suc-AFK-AMC were used in enzymatic activity assays, and higher activities with these substrates were found in the cytosolic fraction (392 ± 30 µmol·min^−1^ mg of protein^−1^ and 252 ± 20 µmol·min^−1^ mg of protein^−1^, respectively) than in the membrane fraction (53 ± 5 µmol·min^−1^ mg of protein^−1^ and 63 ± 6 µmol·min^−1^·mg of protein^−1^, respectively, see Figure 1). The specificity was confirmed by inhibition assays in the presence of selective inhibitors. The proteinase activity against Z-FR-AMC and Suc-AFK-AMC was more strongly inhibited by aprotinin in the cytosolic fraction (77% and 44%, respectively) than in the membrane fraction (39% and 19%, respectively). PMSF also inhibited the activity against Z-FR-AMC in the cytosolic (63%) and membrane (69%) fractions but not against Suc-AFK-AMC in the membrane and cytosolic fractions (approximately 3%). Interestingly, E-64 only inhibited the enzymatic activity in the cytosolic fraction when Z-FR-AMC was the substrate (61%, see Figure 1).

### 2.2. Gene Transcripts Detected in Promastigotes

**Figure 2:**
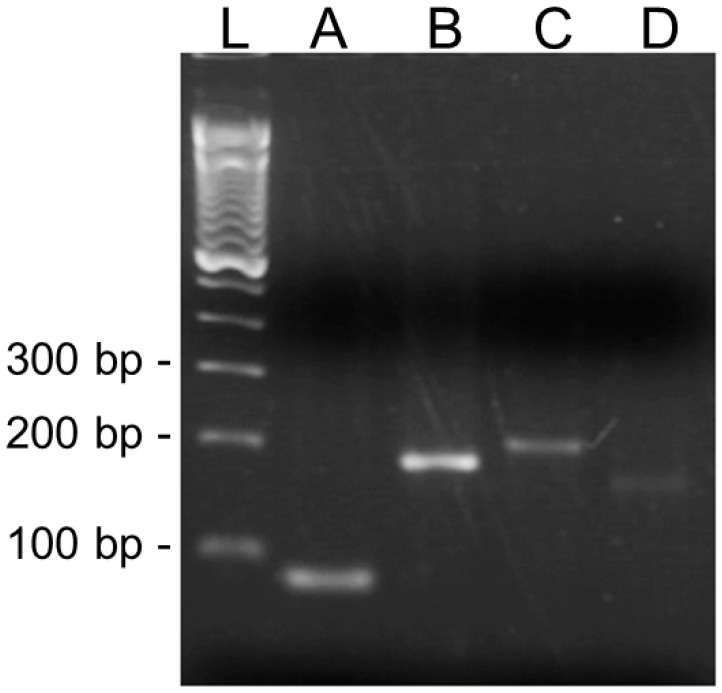
Reverse transcription polymerase chain reaction (RT-PCR) of *Leishmania (V.) braziliensis* genes. The assays were performed with promastigote mRNA samples obtained from parasites in the stationary growth phase. RT-PCR assays were performed for the 40S ribosomal protein S8 (LbrM.24.2160) (**A**), tryparedoxin peroxidase (LbrM.15.1080) (**B**), and two subtilisin (LbrM.13.0860 (**C**) and LbrM.28.2570 (**D**)) genes, and revealed single 98 bp, 166 bp, 200 bp, and 162 bp fragments, respectively, in the tested cDNA samples. Electrophoretic analysis was performed in 2% agarose gels, and a 100 bp DNA ladder (**L**) was used as a molecular weight marker.

In the second step of this study, the expression of *L. (V.) braziliensis* LbrM.13.0860 and LbrM.28.2570 genes was investigated. As expected, the designed primers were specific for the conserved region of the *L. (V.) braziliensis* subtilisin gene and were used to amplify a 200 bp fragment of LbrM.13.0860 and a 162 bp fragment of LbrM.28.2570 from cDNA samples by PCR (Figure 2). The 98 bp 40S ribosomal protein S8 gene (LbrM.24.2160) was included in this study as a constitutive gene expression control. The intensity of the bands for the PCR products indicated more LbrM.13.0860 than LbrM.28.2570 transcripts in the promastigote cDNA samples. Additionally, LbrM.15.1080 transcripts were also observed in promastigotes in the stationary phase, which yielded a 166 pb PCR product.

### 2.3. Prediction of Subtilisin Subcellular Localization

**Figure 3:**
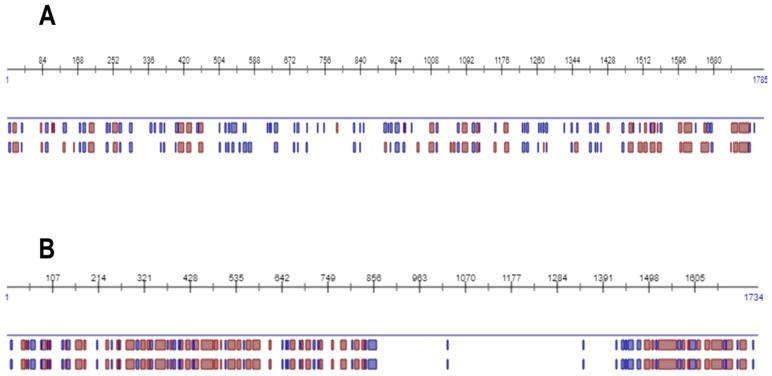
Secondary structure content of *Leishmania (V.) braziliensis* subtilisins. The sequences of the subtilisin LbrM.13.0860 (**A**) and LbrM.28.2570 (**B**) were analyzed by the PredictProtein server. The predicted secondary structures are colored blue (β-strands) and red (α-helices).

**Figure 4:**
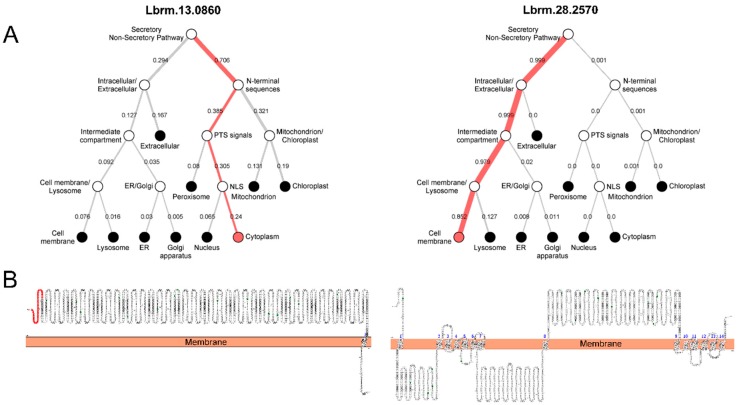
Prediction of the subcellular location of *Leishmania (V.) braziliensis* subtilisins. LbrM.13.0860 and LbrM.28.2570 were predicted to be located in the cytosol and cellular membrane, respectively, by analysis with the DeepLoc-1 (**A**) and Protter (**B**) servers. In (**A**): hierarchical tree of the prediction of proteins located in cellular compartments. The red lines show the most likely paths of the proteins in the cell calculated by the program. In (**B**): the predicted topological structure of both subtilisins showing proteoforms such as N-glycosytation (green) and the presence of a signal peptide (red). Finger-like projections are loops joining N- and C-terminal groups of transmembrane segments. Blue numbers show the amino acid residues inserted into the cell membrane.

In the third step of this study, an in silico analysis was performed to predict the location of *L. (V.) braziliensis* subtilisins (LbrM.13.0860 and LbrM.28.2570), based on their protein structures (Figure 3 and Figure 4). Calculations were performed using the PredictProtein server and indicated that both proteins differ in their secondary structure content as follows: LbrM.13.0860 [β-strand (9.9%), α-helix (15.7%) and loop (74.4%)] and LbrM.28.2570 [β-strand (10.7%), α-helix (26.5%), and loop (62.8%)], (Figure 3).

Differences were also observed in the prediction of the subcellular localizations of these proteins, which was based only on sequence properties by using a deep neural network provided by the DeepLoc-1 server. The accuracy of these analyses was assessed with three *L. (V.) braziliensis* proteins (two with RNA binding capacity (LbrM.25.2210 and LbrM.30.3080) [15], and dipeptidyl-peptidase 3 (LbrM.05.0940) [16], all of which have experimentally defined subcellular locations. The program accurately identified the cellular location of LbrM.25.2210 as the parasite nucleus (0.50) and LbrM.30.3080 and LbrM.05.0940 as the cytoplasm (0.85 and 0.97, respectively, data not shown). After confirming the accuracy of the analysis, the proteins from the LbrM.13.0860 and LbrM.28.2580 genes were predicted to be cytoplasmic (0.24) and membranous (0.85), respectively (Figure 4A).

Data gathered using the Protter server showed that LbrM.13.0860 (Figure 4B) contains mostly free sequences between amino acids 1735 and 1753, which indicate its localization within an organelle or in the cytoplasm. In contrast, the LbrM.28.2570 protein contains transmembrane regions along its entire sequence and is most likely present on the membrane of an organelle or on the surface membrane of the parasite. The conformation of the predicted protein identified amino acid residues with N-type glycosylation in LbrM.13.0860 (*n* = 16) and LbrM.28.2570 (*n* = 15). In addition, a signal peptide sequence was identified in the N-terminal portion (1 to 38 amino acids) of the LbrM.13.0860 protein (Figure 4B).

## 3. Discussion

Serine proteinases from *Leishmania (V.) braziliensis* promastigotes are poorly understood, especially regarding their roles in the adaptation to the environment within an invertebrate host, and biochemical studies related to these enzymes could improve our understanding of this parasite physiology. These enzymes are believed to act in a network of proteins that catalyze the cleavage of peptide bonds to maintain the physiological equilibrium of this parasite [7]. Therefore, studies to identify these enzymes in promastigotes are necessary for understanding the mechanism used by parasites for their adaptation to insect environments. In the present study, it was proposed that serine proteinases are found in the cytoplasm and membrane of *L. (V.) braziliensis* promastigotes, which are parasites responsible for most tegumentary leishmaniasis cases in Latin America [17].

To determine the location of proteinases in *L. (V.) braziliensis* promastigotes, a successful approach involving the fractionation of cellular compartments was applied here. In a broader analysis, subcellular fractions obtained from promastigotes in the stationary phase of growth exhibited serine proteinase activity demonstrated by the hydrolysis of gelatin and were distributed in the cellular compartments of the parasite, such as the cytosol (43 kDa to 170 kDa) and membrane (67 kDa to 170 kDa). Specific enzyme assays confirmed that protein preparations obtained by affinity chromatography contained serine proteinases, due to their ability to cleave highly specific fluorogenic substrates such as Z-FR-AMC, a substrate of cathepsins, kallikrein, and plasmin [18], and Suc-AFK-AMC, a substrate of plasmin [19], both of which contain positively charged residues at the P1 position.

Furthermore, molecular masses found in both fractions agree with previous findings regarding the serine proteinases in *L. (V.) braziliensis* promastigote, which have been described as exhibiting a broad range of molecular weights from 30 kDa to 130 kDa with gelatinolytic activity [20,21]. Nevertheless, it is important to comment that the applied methodology in these two previous studies differs from the strategy used in the present work, which can explain some differences in the serine proteinase profile detected here. In fact, the combination of subcellular fractioning and benzamidine-agarose chromatography was favorable for finding serine proteinase bands not previously detected using detergent fractioning [20] and freeze-thawing, differential centrifugation, and aprotinin-agarose chromatography [21]. In addition, the enzyme profile obtained in this work showed a broader range of molecular masses that is different from the subtilisin profile described for *Bacillus subtilis*, which included molecular masses from 20 kDa to 45 kDa [22,23,24]. However, it is necessary to highlight that the higher molecular masses detected here by gelatinolytic activities are close to the predicted molecular masses for LbrM.13.0860 (~190.8 kDa) in the cytosolic fraction and as LbrM.28.2570 (~188.2 kDa) in the membranous fraction. In addition, this is the first study to report the serine proteinase profile of *L. (V.) braziliensis* promastigotes purified based on their affinity to benzamidine.

The analysis of the serine proteinase subcellular locations performed here was an important strategy, because a protein’s function, as well as its interactive networks, depends on the cellular location of each protein component in the respective network. Thus, during this work, it was opportune to perform the in silico characterization of *L*. (*V*) *braziliensis* subtilisin cellular location. These analyses yielded interesting findings that need some explanation. We compared the performance of the DeepLoc-1 and Protter model prediction algorithms and found that both models significantly predicted the LbrM.13.0860 and LbrM.28.2570 as cytosolic and membrane proteins, respectively. Furthermore, it is important to emphasize that both methods are sequence-based methods that do not rely on annotation information from homologous proteins [25,26].

Since these algorithms indicated LbrM.28.2570 to be a membrane protein with intracellular and extracellular regions, the arrangement of the catalytic site of this enzyme requires additional comments. Comparative sequence analysis of LbrM.13.0860 and LbrM.28.2570 genes (Supplementary Material Figure S1) showed 16.1% sequence identity based on the catalytic site of LbrM.13.0860 gene in the S8 domain. While LbrM13.0860 gene has well-defined catalytic site residues (Aspartic Acid 97, Histidine 130, Asparagine 273 and Serine 339), the catalytic residues of the LbrM28.2570 gene have not yet been defined. However, by aligning the S8 domain of both genes, it was possible to find that LbrM13.0860 was aligned with the extracellular and intracellular regions of LbrM.28.2570, showing that both regions have the potential to form a catalytic site similar to described for LbrM13.0860 gene. Different quantities of these residues are distributed at different positions in the intracellular region [aspartic acid (*n* = 7), histidine (*n* = 3), asparagine (*n* = 7) and serine (*n* = 11)] and the extracellular region [aspartic acid (*n* = 12), histidine (*n* = 2), asparagine (*n* = 10) and serine (*n* = 15)].

The cellular locations of these enzymes in *L. (V.) braziliensis* agrees with the possible action of these enzymes in the parasite [7], since contact with the host and some metabolic pathways occurs at physiological pH. This can be explained by the catalytic site of serine proteinase, which is part of a charged network involving the amino acid residues in the catalytic triad. It is possible that this charged network can be activated by subtilisin from this protozoan in its environment, as proposed for bacterial subtilisin [27,28,29]. Since a charged network can influence the activity of promastigote serine proteinases in the spaces within the host’s body, the catalytic site in these enzymes must have an organization favorable for these activities. Thus, it is possible that the charged network containing the catalytic site of LbrM.28.2570 is dependent on the final fold of this protein, which in turn is dependent on the chemical environment in the different hosts of the parasite.

The results of the enzymatic activity in this work corroborated the predictive calculations of the subcellular localizations of the subtilisins in *L*. (*V*) *braziliensis* and are in agreement with one of the functions described for these enzymes, namely, processing TXNPx in amastigotes [30,31,32,33]. In addition, due to the presence of LbrM.15.1080 transcripts in promastigotes in the stationary growth phase in culture, and the fact that *Leishmania (Leishmania) amazonensis* promastigotes expressed TXNPx during their growth in culture [34], it is plausible that subtilisins can also act as maturases for promastigote proteins.

Additionally, an interesting finding of this work was the inhibition profile of E-64, an irreversible and selective inhibitor of cysteine proteinases that also inhibits serine proteinases, except for trypsin-like proteinases [35]. The inhibitory effect observed on the cytosolic fraction with Z-FR-AMC substrate suggested that some of the serine proteinases in the cytosol have a catalytic mechanism similar to that of cysteine proteinases. This finding can be explained by the similarity of the catalytic mechanisms of both proteinases, which are influenced by pH change driven by pH-sensing residues [36]. Another explanation for this finding is the possibility that benzamidine affinity chromatography retained cysteine proteinases from the protein preparations due to hydrogen bonding residues arranged without tension, and the formation of an oxyanion hole resembling the active site of a serine proteinase [37].

Finally, in this work, we described the transcription of genes for subtilisins in promastigotes by RT-PCR. Although these results suggested that both LbrM.13.0860 and LbrM.28.2570 gene transcripts are produced by stationary phase promastigotes under culture conditions, the specific PCR amplification product profiles detected on an agarose gel suggested a quantitative difference in the expression of both genes. It is likely that these genes may be expressed alternately during the culture timeline, but additional assays are required to prove this hypothesis.

## 4. Materials and Methods

*Reagents*—Detergents [Tween 20, Triton X-100 (TX-100), and 3-[(3-cholamidopropyl)-dimethylammonium]-1-propanesulfonate (CHAPS)], gelatin, bovine serum albumin (BSA), penicillin, proteinase inhibitors [*trans*-epoxysuccinyl-l-leucylamido(4-guanidino)butane (E-64), phenylmethanesulfonyl fluoride (PMSF) and aprotinin], fluorogenic peptide substrates [*N*-benzyloxycarbonyl-l-phenylalanyl-l-arginine 7-amino-4-methylcoumarin (Z-FR-AMC) and *N*-succinyl-l-alanine-l-phenylalanine-l-lysine 7-amino-4-methylcoumarin (Suc-AFK-AMC); ε= 1.78 × 10^4^ M^−1^ cm^−1^], a ProteoSilver™ Silver Stain Kit, and Nancy-520 DNA gel stain were purchased from Sigma Aldrich Chemical Co. (St. Louis, MO, USA). A HiTrap^®^ Benzamidine Fast Flow column (HiTrap Benzamidine; 1.5 × 2.5 cm) was purchased from GE Healthcare. Fetal calf serum (FCS) was purchased from Cultilab S/A (Campinas, SP, Brazil). The electrophoresis reagents were purchased from Bio-Rad Laboratories Inc. (Hercules, CA, USA). The full-range Rainbow^TM^ kit (12 to 225 kDa) was purchased from GE Healthcare Life’s Sciences (Little Chalfont, UK). Amicon Centriprep YM-10 filter devices were purchased from Millipore (Burlington, MA, USA). TRIzol^®^ RNA Isolation Reagent (TRIzol), RNase Henzyme, DEPC-treated water, deoxyribonucleotide phosphate solution (dNTPs), a SuperScript III First-strand cDNA Synthesis Kit (SuperScript III kit), Platinum Taq DNA Polymerase (DNA Polymerase), and Taq Platinum PCR buffer were purchased from Invitrogen Life Technologies (Carlsbad, CA, USA). A RNeasy Mini Kit and RNase OUT enzymes were purchased from Qiagen (Hilden, Germany). A Wizard SV Gel Kit and PCR Clean-Up System were purchased from Promega Corporation (Madison, WI, USA). All other reagents were of analytical grade or better.

*Parasite cultures*—*Leishmania* *(Viannia) braziliensis* (MCAN/BR/1998/619) promastigotes were maintained at 28 °C in Schneider’s medium pH 7.2 (containing 1 mM l-glutamine, 10% FCS, 100 IU/mL penicillin, and 100 μg/mL streptomycin), with frequent sub passages to maintain the parasites in the logarithmic growth phase. Parasites from the stationary phase of growth after four days of culturing were used in subsequent experiments.

*Subcellular fractionation*—Subcellular fractions enriched with membrane and cytosolic proteins were obtained by centrifugal fractionation as previously described [38,39]. Briefly, promastigotes (10^8^ cells/mL) were washed twice (3800× *g*, 4 °C, 10 min) in PBS pH 7·0, followed by two more wash cycles (3800× *g*, 4 °C, 10 min) in 10 mM Tris-HCl pH 7·2 buffer containing 1 M NaCl, 0·2 M K_2_HPO_4_, and 0·5 M MgCl_2_. The pellet (10 mL/g promastigotes) was then resuspended in 10 mM Tris-HCl, pH 7·5, containing 0·05 M sucrose, and disrupted in 1% CHAPS using a Dounce-type homogenizer with 0·25 M sucrose. After centrifugation (4300× *g*, 4 °C, 10 min), the resultant supernatant was centrifuged (12,000× *g*, 4 °C, 15 min), and the supernatant was centrifuged again (35,000× *g*, 4 °C, 45 min) to obtain a new pellet that contained the membrane fraction. The supernatant of the last centrifugation step was the cytosolic fraction.

*Benzamidine affinity chromatography*—Serine proteinase-enriched fractions were obtained from soluble subcellular protein preparations (30 mg/mL). The protein preparations were resuspended in buffer (10 mM Tris-HCl, pH 7.5, containing 1% CHAPS) and passed through a HiTrap Benzamidine column that had been previously equilibrated with binding buffer (0.05 M Tris-HCl, 0.5 M NaCl, pH 7.5). The column was washed with the same buffer to remove unbound proteins, and the bound proteins were retrieved using elution buffer (0.05 M glycine, pH 3.0) and preserved in an appropriate buffer (1 M Tris-HCl, pH 9.0). The eluted proteins were concentrated and dialyzed against buffer (10 mM Tris-HCl, pH 7.5, containing 1% CHAPS) in an Amicon Centriprep concentrator for further use.

*Protein determination*—Protein concentrations were determined by colorimetric assay as previously described [40], using BSA as a standard protein.

*Zymographic assays*—The protein fraction (15 μg) was subjected to electrophoresis under reductive conditions using 12% acrylamide gels copolymerized with 0.1% gelatin [41]. Following electrophoresis, the gels were washed (4 °C, 1 h) in 0.1 M Tris-HCl pH 7.5 containing 2.5% Triton X-100 and then incubated (37 °C, 12 h) in the same buffer. Proteinase bands were revealed by staining with Coomassie Brilliant Blue R-250. The molecular masses of the proteinase bands were calculated by comparison with the mobility of molecular mass standards (12 to 225 kDa).

*Proteinase activity in solution*—The proteinase activities of the serine proteinase-enriched fraction (5.0 μg total protein) were accessed in activation buffer (10 mM Tris-HCl pH 7.5), at a final volume of 60 μL using specific fluorogenic peptide substrates of serine proteinases (Z-FR-AMC and Suc-AFK-AMC at 0.1 mM). Samples were incubated (37 °C, 60 min), and the variance in the relative fluorescence was monitored with a Molecular Devices SpectraMax spectrophotometer (Gemini XPS). Additionally, inhibition assays were performed by the addition of PMSF (1 mM), aprotinin (5.0 μg) or E-64 (10 μM). The substrate cleavage rate was defined as follows: v = Δs/Δt, where v = velocity, Δs = substrate concentration variation and Δt = total reaction time [42]. The self-degradation of the fluorescent peptide substrate was controlled throughout the assay to avoid incorrect readings. The enzymatic activity is expressed as µmol min^−^^1^·mg of protein^−1^.

*In silico analysis of subtilisin sequences*—The sequences of the LbrM.13.0860 and LbrM.28.2570 subtilisins were analyzed based on their structures by using automatic servers accessed in free online platforms. Data on the secondary structures of both protein sequences were obtained by using the PredictProtein server (https://www.predictprotein.org/), [43]. The subcellular localization of proteins was assessed using the DeepLoc-1.0 server (http://www.cbs.dtu.dk/services/DeepLoc/), a eukaryotic protein subcellular localization predictor based on a hierarchical tree calculation used to predict the location of both proteins in cellular compartments. The data were generated from the percentage probability of the protein in each compartment of a eukaryotic cell [25]. The Protter server (http://wlab.ethz.ch/protter/start/) was used to visualize proteoforms [26]. The models were proposed based on the predicted protein products of both genes, according to the characteristics of their amino acid residues.

*Selection of sequences and primer design*—The primers were designed for this study based on the *Leishmania* (*Viannia*) *braziliensis* subtilisin (LbrM.13.0860: sense-5′ ATCTGGCGATTTCTCCCTTT 3′ and antisense-5′ GAGCTAACACCAG 3′; LbrM.28.2570: sense-5′ CACTGCGCTCCACATACACT 3′ and antisense-5′ GCCTTCATTCGAGCTACAGG 3′) and tryparedoxin peroxidase (LbrM.15.1080: sense-5′ CTCTGTGGACAGCGAGTACG 3′ and antisense-5′ TGGGGTCGATGATAAAGAGG 3′) sequences recorded in the Genedb database (http://www.genedb.org). The primers were designed using the online software Primer3 v. 0.4.0 (http://frodo.wi.mit.edu/primer3/), with all parameters set to default except the product size range, which was adjusted to 80–200 base pairs (bp). Additionally, primers complementary to the 40S ribosomal protein S8 gene (LbrM.24.2160: sense-5′ CGACTTGGATGCGGGGA 3′ and antisense -5′ GGCGAAGCCTTGTTCACG 3′) were used [44,45]. The primers were synthesized by Invitrogen Brazil at a concentration of 50 nM and purified by desalting.

*RNA extraction and cDNA synthesis*—Promastigotes in the stationary phase (10^7^/mL) were lysed (1 mL TRIzol containing 200 μL chloroform). RNA extraction was performed using the RNeasy Mini Kit according to the manufacturer’s instructions. The sample was incubated (25 °C, 5 min) and centrifuged (10,000× *g*, 4 °C, 18 min). The supernatants containing RNA were mixed with ethanol (400 μL) and transferred to the column provided in the RNeasy Mini kit and then centrifuged (7200× *g*, 4 °C, 30 s). After that, the column was washed by centrifuged (7200× *g*, 4 °C, 30 s) once with RW1 buffer (700 μL) and then twice with RPE buffer (500 μL). Then, the samples were recovered in 30 μL DNAse free water (1 U/μL), and concentrations were measured by spectrophotometry at 260 nm. The RNA integrity was evaluated by 1.2% (*w*/*v*) denaturing agarose gel electrophoresis stained with Nancy-520 DNA gel stain. cDNA synthesis was performed using the SuperScript III First-Strand Synthesis System for RT-PCR (1 μg of total RNA).

*Reverse Transcription Polymerase Chain Reaction (RT-PCR)*—Primers complementary to the LbrM.24.2160, LbrM.13.0860, and LbrM.28.2570 genes were used for PCR under the following reaction conditions: 1 mM Tris-HCl, pH 8.3, 50 mM KCl, 4 mM MgCl_2_, 2.5 mM of each dNTP, 0.5 µM of each primer and 1 U Taq DNA polymerase, together with 500 ng of cDNA and adjusted to a final volume of 25 μL. The amplification cycle was performed as follows: one step (94 °C, 5 min) followed by 30 cycles (95 °C, 45 s; 60 °C, 45 s; 72 °C, 90 s) and a final elongation step (72 °C, 7 min) using a PTC-100 Thermal Cycler (Applied Biosystems, Foster City, CA, USA). The amplified products (5 µL) were evaluated by 1.2% (*w*/*v*) agarose gel electrophoresis and stained with ethidium bromide.

## 5. Conclusions

The results presented here bring new insight into the serine proteinases of *L. (V.) braziliensis* promastigotes and provide evidence of the active production of transcripts for both subtilisin (LbrM.13.0860 and LbrM.28.2570) and TXNPx (LbrM.15.1080) genes. Benzamidine used during the purification step accessed serine proteinases from the cytosol and membrane with molecular masses close to the predicted molecular masses of *L. (V.) braziliensis* subtilisins. Based on the structural features of their sequences, it was postulated that part of the enzymatic activity detected in the cytosolic fraction was due to the LbrM.13.0860 gene expression product, because of the predominance of hydrophilic amino acids, while the activity in the membrane fraction was partly due to the LbrM.28.2570 gene expression product, because most of its amino acids are hydrophobic. These findings suggest that promastigotes inhabiting sand flies express serine proteinases that may contribute to the maintenance of this parasite’s lifestyle at physiological pH, in the cytosol, and on the external face of the parasitic membrane.

## Supplementary Materials

Supplementary materials are available online at https://www.mdpi.com/1422-0067/20/6/1315/s1.
