# Supplementary material for: Serine Proteinases in Leishmania (Viannia) braziliensis Promastigotes Have Distinct Subcellular Distributions and Expression
Source: Int J Mol Sci. 2019 Mar 15;20(6):1315. doi: 10.3390/ijms20061315 (PMC6471289; doi:10.3390/ijms20061315)
Supplement: Supplementary file 1 [file ijms-20-01315-s001.pdf]

Supplementary material

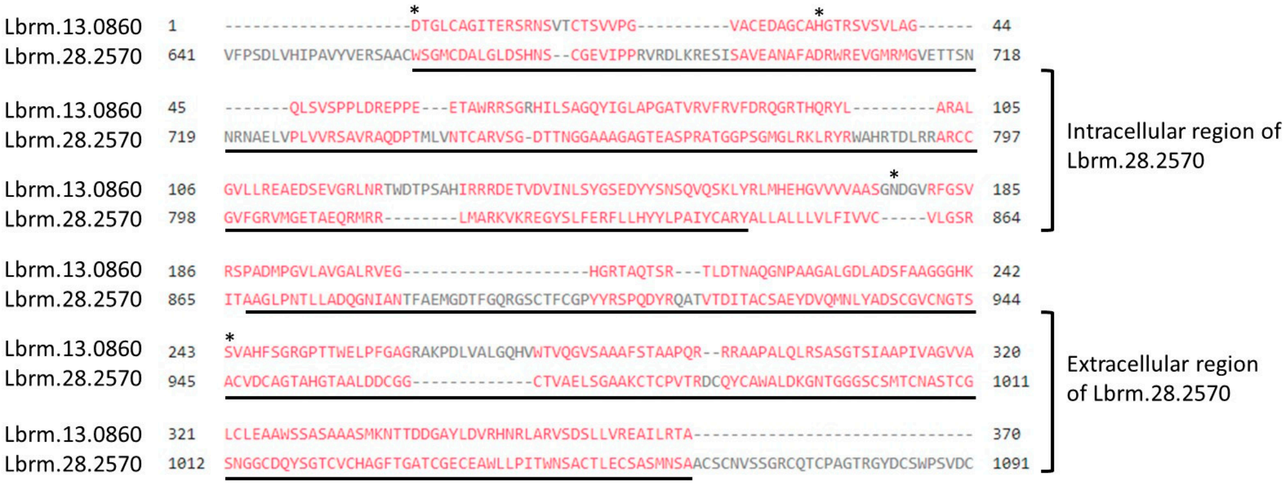

Figure S1. Comparative sequential analysis of the S8 domain of the LbrM.13.0860 and LbrM.28.2570 genes. Sequence analysis was performed on the BLAST server (https://blast.ncbi.nlm.nih.gov). In the figure is shown the alignment of the region corresponding to the S8 domain based on the amino acid residues of the catalytic site (*) of the LbrM.13.0860 gene. In the Lbrm28.2570 gene, amino acid residues with potential for active site formation can be detected in the intracellular and extracellular regions: Aspartic (D), Asparagine (N), Histidine (H) and Serine (S).
